# The kinetic model for slow photoinduced electron transport in the reaction centers of purple bacteria

**DOI:** 10.1186/s11671-016-1502-x

**Published:** 2016-06-07

**Authors:** T. V. Serdenko, Y. M. Barabash, P. P. Knox, N. Kh. Seifullina

**Affiliations:** Department of Physics of Biological Systems, Institute of Physics NAS Ukraine, Prospect Nauky, 46, 03028 Kyiv, Ukraine; Department of Biophysics, Biology Faculty of the M.V. Lomonosov Moscow State University, Leninskie Gory, 1, 119991 Moscow, Russia

**Keywords:** Reaction center, Purple bacteria, Photoinduced electron transport, Two-level kinetic model, Electron-conformational states of biological macromolecules, 73.20.-f, 87.15.hp, 87.15.ht

## Abstract

The present work is related to the investigation of slow kinetics of electron transport in the reaction centers (RCs) of *Rhodobacter sphaeroides*. Experimental data on the absorption kinetics of aqueous solutions of reaction centers at different modes of photoexcitation are given. It is shown that the kinetics of oxidation and reduction of RCs are well described by the sum of three exponential functions. This allows to suggest a two-level kinetic model for electron transport in the RC as a system of four electron-conformational states which correspond to three balance differential equations combined with state equation. The solution of inverse problem made it possible to obtain the rate constant values in kinetic equations for different times and intensities of exciting light. Analysis of rate constant values in different modes of RC excitation allowed to suggest that two mechanisms of structural changes are involved in RC photo-oxidation. One mechanism leads to the increment of the rate of electron return, another one—to its drop. Structural changes were found out to occur in the RCs under incident light. After light was turned off, the reduction of RCs was determined by the second mechanism.

## Background

**Fig. 1:**
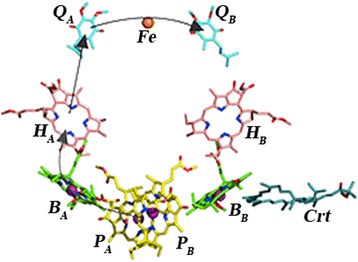
The photosynthetic reaction center from *Rhodobacter* (*Rba.*) *sphaeroides* [8]

The membrane protein-pigment complexes of photosynthetic reaction centers (RCs) are macromolecular systems, which are used for studying the physical mechanisms of electron and proton transport in biological structures, the role of molecular dynamics in highly efficient processes of charge transport and control, and electrostatic and dielectric properties of proteins. This properties are very important for protein functioning. Under photoexcitation, the RCs provide electron and ion transport and ATP synthesis [1–6]. RC structure is well known, and J. Deisenhofer, R. Huber, and H. Michel won the Nobel Prize in Chemistry in 1988 for their research in this field. The RC includes the following cofactors: four bacteriochlorophylls (P, B), two bacteriopheophytins (H), two quinones (Q_A_ and Q_B_), and one high-spin atom of iron (Fe^2+^). Photosynthetic RC contains three membranous subunits L, M, and H with molecular weights of 19 (light), 22 (medium), and 28 × 10^3^ (heavy), respectively. L and M subunits are very similar (Fig. 1).

During RC photoexcitation, a light quantum results in excitonic excitation of the bacteriochlorophyll molecules. Its decay causes electron transport through the bacteriochlorophyll monomer to the bacteriochlorophyll dimer (P_A_–P_B_). This electron in less than 10^−12^ s passes through the bacteriochlorophyll monomer B_A_ onto the pheophytin H_A_, overcoming the distance of ~3 Å. Then, in ~10^−10^ s, electron moves from the pheophytin H_A_ onto the quinone Q_A_, and the rate of this transfer does not depend on the temperature. Next, the electron transfer onto the quinone Q_B_ (secondary acceptor) occurs in ~10^−4^ s [6]. The electron overcomes a total distance of ~40 Å. At each cofactor of this chain of electron transfer, the stability depends on the structural and dynamic setup of the molecular complex of RC. In the case of separate RCs consisting of photosynthetic membranes and absence of exogenous electron donors, the electron slowly returns from photo-reduced acceptors back to the oxidized bacteriochlorophyll after the exciting light is switched off.

The accumulated experimental data prove the fundamental necessity of adequate physical description of the electron transport processes in photosynthetic RCs, considering conformational fluctuations in macromolecular system, the possibilities of its evolution in conformational microstates, and the probability of structural rearrangement of the protein at various stages of electron transport [4, 7, 8]. The conformational dynamics plays a key role in the effective time stabilization of electron in the quinone acceptor chain in photosynthetic RCs of purple bacteria [9–12]. The important functional significance of effective work of this section of electron transport chain is primarily determined by the fact that exactly this section connects very fast initial stages of light-induced charge separation with significantly slower ones, controlled by diffusion reactions of transport of reduction equivalents into the photosynthetic membrane. As a result, two molecules with quinones Q_A_ and Q_B_, integrated into the structure of purple bacteria’s RCs, are actively involved in the conjugated electron-proton transport processes that lead to the formation of proton gradient across the photosynthetic membrane, needed for ATP synthesis. Basing on specific experimental data, one of the first models for conformational transitions in the photosynthetic RCs was built, which accounts for the direction and rate of electron transport in the acceptor part of RC [13]. A detailed analysis of non-monoexponential kinetics of dark recombination of light-oxidized photoactive bacteriochlorophyll and reduced quinone acceptors, the dependence of characteristic time of the process on the regimes of light activation, and temperatures of the samples allows to present the purple bacteria’s RC as a system capable of self-organization. The repetition of elementary acts of electron transport leads to the emergence of new conformational sub-states that promote effective stabilization of the photoelectron in the acceptor part of RC, preventing the functionally disadvantageous recombination of photoproducts [14, 15].

Currently, it is evident that during the electron transmembrane transport from a photoactive pigment to the final quinone acceptor in RC electron localization at each cofactor of this transfer chain is provided with structurally dynamic organization of RC complex. Obviously, the development of long-life states with charges divided between the P dimer and quinone acceptors is related to the generalization of structural and dynamic changes in the RC. It is not possible to localize definitely the changes in RC structure, which result in the progressive slowdown of reverse reduction of P in darkness after its oxidation caused by a prolonged exposure to constant light [16, 17]. Further detailed kinetic analysis of the non-monoexponentiality of the kinetics of interaction of photoactive bacteriophyll with quinone acceptor under conditions of prolonged activation of RC samples by light of different intensities can provide additional information on the structural and dynamic changes in the RC, which are related to electron transport between the P dimer and quinone acceptors. This is the subject of the present work.

## Methods

For RC investigation, a PC-controlled two-channel spectrometer was developed; the spectrometer provided measurements of optical absorption kinetics (*λ* = 865 ± 10 nm) in the range of 0 ÷ 1 ± 0.0005, using light pulses (5 kHz) of low intensity (0.2 μW/cm^2^), and photoexcitation of RC by light (*λ* = 870 ± 50 nm) with intensities from 0 up to 10 mW/cm^2^. Channels are arranged perpendicular to each other in the space. Measuring channel for optical absorption includes an AC voltage amplifiers with a synchronous detector tuned to a frequency of 5 kHz. Measuring channel for photoexcitation intensity involved DC voltage amplifiers. Compensation unit of passing photoexcitation pulses fronts to measuring absorption channel was developed. It is possible to achieve a level of reciprocal signal suppression to above 60 decibels. The resolution of measurements was 0.01 s. The measuring cuvette had dimensions of 3 × 1 × 2.5 cm and wall thickness of 2 mm.

The investigations were conducted on RCs separated from wild-type bacteria *Rhodobacter sphaeroides*. The bacteria’s chromatophores were stabilized by LDAO detergent; RCs were separated from other membrane components by column chromatography with hydroxyapatite. Separated RCs were suspended in a 0.01-M Na-P buffer with a pH of 7.2, which contained 0.05 % LDAO [18].

The prepared RC solution of ~10^−6^ M concentration was kept in darkness for 12 h at room temperature. The absorption by RC solution was implemented by means of its illumination by light pulses of different durations and intensities. The absorption measurements allow to judge about the electron transport. The recovery of RC absorption to the values inherent to dark environment does not ensure the completion of conformational changes in the RCs and their return to the thermo-adapted state.

**Fig. 2:**
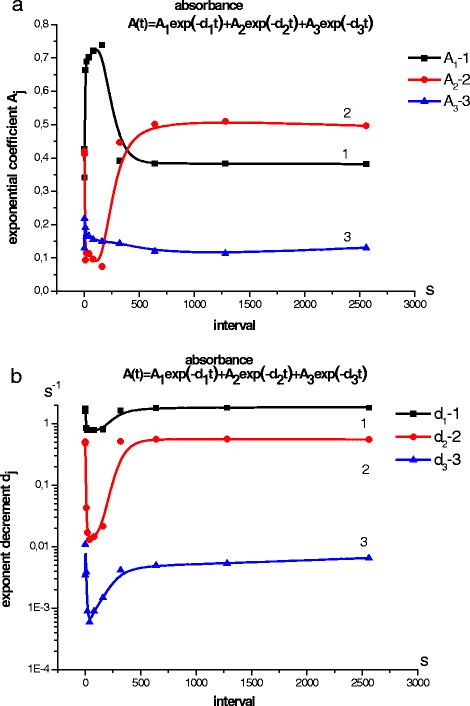
**a** Exponential coefficient change of the exponential component of kinetic curve of RC reduction. **b** Change of decrement (exponent decrement) of the exponential component of kinetic curve of RC reduction

The applied protocol for measuring the absorption kinetics of RC solutions for the series of measurements provided that after the excitation light was switched off and the absorption reached the value inherent to darkness, the light for RC excitation in the next set of measurements was switched on with a delay of 1500 s. For the delays of 1500 s the kinetics of absorption on RCs excited by two successive light pulses (100 s) were measured. The interval between the pulses varied from 0 up to 2500 s. Figure 2 shows how the parameters A_j_ and d_j_ of RC reduction process changed after the second excitation pulse was switched off, for different intervals between the excitation pulses ($A={\displaystyle {\sum}_{i=1}^n{A}_i}{e}^{-{d}_it}$).

It is seen that at the interval between the exciting pulses more than 700 s the parameters A_j_ and d_j_ of RC reduction process did not change after switching off the second pulse, and the pulses were independent from each other. Therefore, the measurement protocol of the kinetics of electron transfer in RC consists of recording the absorption value of RC in darkness, and photoexcitation of RC by light of a given intensity and duration, with recording the absorption value of RC until it reaches the magnitude corresponding to darkness, and a delay for 1500 s. Only in such case, the transition to RC photoexcitation by light with other parameters is possible.

## Results and discussion

**Fig. 3:**
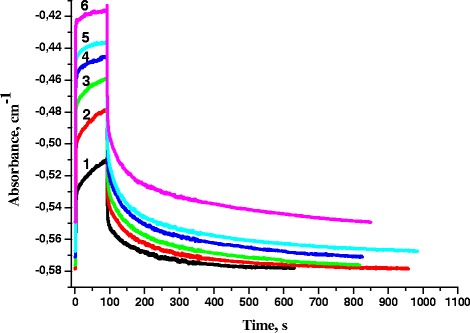
Kinetics of absorbance changes at variable light intensity: *1*—0.2 mW/cm^2^, *2*—0.5 mW/cm^2^, *3*—1 mW/cm^2^, *4*—2 mW/cm^2^, *5*—4 mW/cm^2^, *6*—6 mWcm^2^; illumination time 90 s

**Fig. 4:**
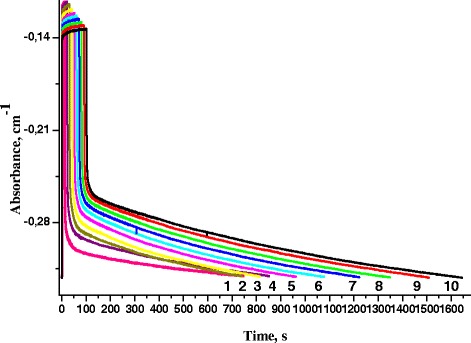
Kinetics of absorbance changes at different illumination time: *1*—10 s, *2*—20 s, *3*—30 s, *4*—40 s, *5*—50 s, *6*—60 s, *7*—70 s, *8*—80 s, *9*—90 s, *10*—100 s; excitation light intensity 6 mW/cm^2^

Using the above mentioned protocol, the kinetics of photoexcitation and relaxation of RCs of photosynthetic bacteria was investigated in the following photoactivation modes: at constant illumination time and discretely variable intensity of photoexcitation (Fig. 3), and at a given intensity of exciting light and different exposure times (Fig. 4).

As seen from Figs. 3 and 4, the kinetics of illumination and darkening of RC solution is depended on the intensity and time of sample illumination. It is seen that the recovery of absorption was delayed with increasing intensity and exposure time. The electron stays longer at Q_B_ with increasing exposure time and illumination intensity, and it is more difficult for it to return to the donor. This may indicate the influence of polarization effects on the internal processes of molecular electron transport.

Nowadays, a two-level scheme is used in the literature to describe the slow processes of electron transport in the RCs. According to this model, the RC is in the ground state (1) when the electron is localized at the donor. After the absorption of photon, the RC passes into an excited state and the electron moves to the acceptor (state (2)). The process kinetics in this model are given by rate constants *k*_12_ and *k*_21_ for direct and inverse transition, respectively; *x*_1_ and *x*_2_ are the probabilities to find the electron in state (1) and (2), respectively. The kinetics of processes, which occur in the RC under light illumination, are described by differential equations with constant coefficients. The multi-level schemes are also described by a system of differential equations with constant coefficients. These systems of equations have exponential solutions. Therefore, the time dependence of the coefficient of optical absorption can be written as an expansion comprised of a certain number of exponential terms: $A={\displaystyle {\sum}_{i=1}^n{A}_i}{e}^{-{d}_it}$, where *A*_*i*_ is the weighting factor and *d*_*i*_ is the logarithmic decrement measured in s^−1^. A special procedure for approximation was developed. The decrement *d*_1_ was chosen in the range of 0.0001…100. The optimal value of weighting factor *A*_1_ at the given *d*_1_ was calculated to obtain the minimal value of the standard deviation of approximation curve from experimental data. Then these values were used for calculating *A*_2_ and *d*_2_ for the second term. Next, *A*_1_ and *d*_1_ were selected for calculated *A*_2_ and *d*_2_. The iterations were stopped as soon as the minimum was reached. Then the procedure was repeated for *A*_3_ and *d*_3_, and so on. The program determined the number of terms, providing the minimal error. In our case, the experimental curves of kinetics of RC electron transport are well approximated by the sum of three exponential terms with constant decrements. For this case, the system with two states is not appropriate as it has a solution with only one exponential term. Therefore, a two-level RC model with four electron-conformational states characterized by constant rates of electron transport (coefficients *k*_*ij*_) was chosen. The investigation of the system with four states with constant coefficients allows to obtain *k*_*ij*_ values for different photoexcitation modes. The model of electron transport with four states of RC system is described by the following system of three differential equations of kinetic balance with constant coefficients and state equation:

$$ \frac{d{x}_1}{dt}=-\left({k}_{12}+{k}_{13}+{k}_{14}\right)\cdot {x}_1+{k}_{21}{x}_2+{k}_{31}{x}_3+{k}_{41}{x}_4 $$,

$$ \frac{d{x}_2}{dt}={k}_{12}{x}_1-\left({k}_{21}+{k}_{23}+{k}_{24}\right)\cdot {x}_2+{k}_{32}{x}_3+{k}_{42}{x}_4 $$,

$$ \frac{d{x}_3}{dt}={k}_{13}{x}_1+{k}_{23}{x}_2-\left({k}_{31}+{k}_{32}+{k}_{34}\right)\cdot {x}_3+{k}_{43}{x}_4 $$,

*x*_1_ + *x*_2_ + *x*_3_ + *x*_4_ = 1.

Substitution of variables allows to obtain a homogeneous system:

$$ \begin{array}{l}{y}_1={x}_1+{h}_1,\\ {}{y}_2={x}_2+{h}_2,\\ {}{y}_3={x}_3+{h}_3,\end{array} $$

and we can find the general solution of the system:

*X*_*i*_(*t*) = *C*_*i*,1_ exp(−*d*_1_*t*) + *C*_*i*,2_ exp(−*d*_2_*t*) + *C*_*i*,3_ exp(−*d*_3_*t*),

where *C*_1_, *C*_2_, and *C*_3_ are the constants determined by the initial conditions of oxidation: *X*_1_(0) = 1, *X*_2_(0) = 0, *X*_3_(0) = 1, *X*_4_(0) = 1. For the reduction process *X*_1_(0), *X*_2_(0), *X*_3_(0), and *X*_4_(0) are equal to the values of *X*_1_(*t*_exp_), *X*_2_(*t*_exp_), *X*_3_(*t*_exp_ ), and *X*_4_(*t*_exp_) during oxidation, respectively. The decrements are expressed through certain relations between *k*_*ij*_*.*

**Fig. 5:**
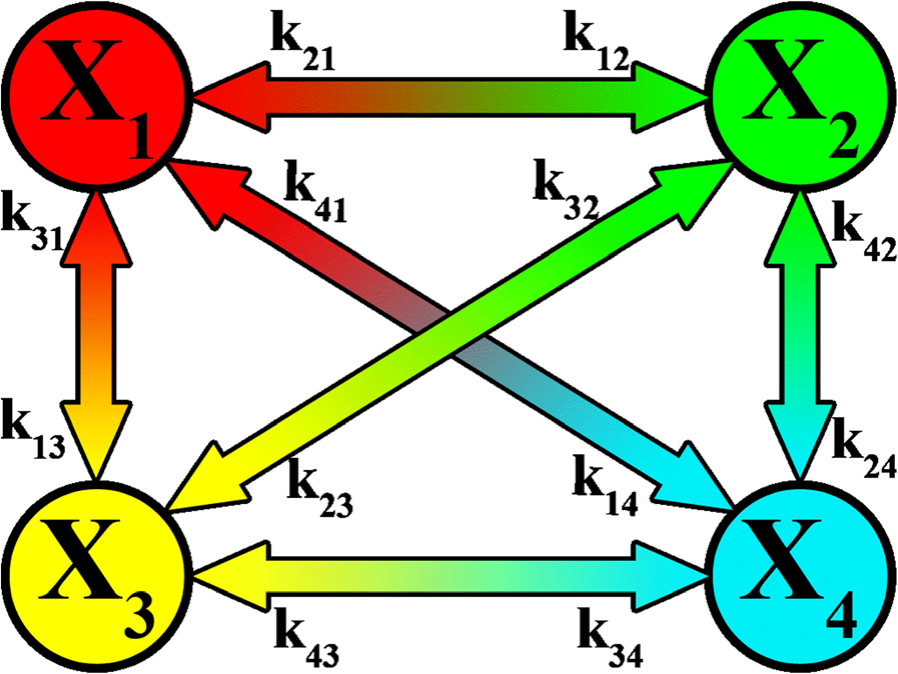
Four-state model of RC

The physical meaning of the electron transport model is that the system can be described by four electron-conformational states (with 12 rate constants) which can change each other (Fig. 5). The electron-conformational interactions in RC occur when the electron gets into the polarized medium formed by the groups of amino-acid residues around the secondary acceptor Q_B_. Thus, the medium is polarized, and a self-consistent field equivalent to the potential well for electron is formed. The polarization deformation can lead to a state which is separated from the initial one by an energy barrier. In biopolymers, the polarization leads to the system shift along the conformational degrees of freedom towards the minimum on the surface that describes the potential energy of the nuclear subsystem. It is considered that the internal dynamics of proteins is characterized by limited diffusion. This means that the conformational movement (one step = 10^−10^ m) which can be spread throughout the protein occurs in small random steps by means of local displacements of atomic groups around their equilibrium positions. This requires a certain local rearrangement of short-range neighbors and overcoming the activation barriers. Typical values of limited diffusion coefficient under normal conditions are of the order of 10^−10^ cm^2^/s, that corresponds to the effective viscosity of protein globule of the order of hundreds poises. Such rearrangements are very important for the adjustment of the configuration of proteins and binding substrates to the chemically reactive states [13].

In the actual RC photosynthetic system the equilibrium conformations of molecules in the presence and absence of an electron can vary greatly due to these electron-conformational interactions. After electron capturing, the acceptor molecule in RC is in a non-equilibrium conformation that slowly relaxes to equilibrium, passing from one local minimum to another on the curve of potential energy. This forms the dynamic picture of the evolution of structural changes, characterized by transfer rates changing with time.

Two models can be considered:

1. A system in which the RC ensemble consists of three fractions, and under illumination some fractions are more amenable to structural changes than other. Thus, the RC ensemble is characterized by three additional states.
2. A system in which all RCs in the darkness adapted state are equivalent, and under illumination, this system can be found with certain probabilities in state 2, state 3 or state 4, and shift between them. This is a two-level system with electron being either at the donor or at acceptor, and it is characterized by four electron-conformational states with different occupancies.

Let us consider the first model: the relaxation times of RCs can significantly differ (by 2–3 orders). It is unlikely in the living bacteria. Besides, in this case, it is difficult to imagine that different fractions can interchange, because the kinetics of reduction in RCs subjected to different conditions of photoexcitation have different sets of exponential functions. Hence, we will use the second model in which all RCs are similar. As a result of excitation, certain amounts of RCs can pass from the first to the second, third, and fourth electron-conformational states. Each RC can move from one electron-conformational state to another due to electron-conformational interactions. The probabilities of these transitions depend on the occupancies of corresponding states. The magnitudes of the transition rates reflect the interaction between the states.

Basing on the experimental data (the first state occupancy) which can be approximated by an exponential expansion, the inverse problem was solved (for a given solution of differential equations the system parameters were found), and the sets of transfer rates (*k*_*ij*_) were calculated for oxidation process at different modes of photoactivation of RC solution. The optimization process was arranged so that the state number corresponded to the serial number in the calculations. At first, the coefficients *k*_12_ and *k*_21_ at zero values of other parameters were determined. Then the coefficients *k*_13_ and *k*_31_ at fixed values of *k*_12_ and *k*_21_ and zero values of other parameters were found. Next, the coefficients *k*_14_ and *k*_41_ at fixed values of *k*_12_, *k*_21_, *k*_13_, and *k*_31_ and zero values of other parameters were calculated. After that, we returned back to the calculations of the coefficients *k*_12_ and *k*_21_, this time at fixed values of *k*_13_, *k*_31_, *k*_14_, *k*_41_, and zero values of other parameters. Then the coefficients *k*_13_ and *k*_31_ were determined and so on. The calculations were iterated until the difference between the transport rate constants in successive steps was within specified limits. Then the coefficients *k*_12_, *k*_21_, *k*_13_, *k*_31_, *k*_14_, and *k*_41_ were fixed, and the cross-parameters *k*_23_, *k*_32_, *k*_24_, *k*_42_ , *k*_34_, and *k*_43_ were alternately calculated. After that, everything was repeated at fixed values of the cross-parameters. After determination of all 12 transport rate constants, the dispersion of these calculations was found. At the end of optimization, the set of coefficients characterized by minimum dispersion was chosen.

After the oxidation process had been calculated, the reduction process was treated. The data on state occupancies at the moment of light turning off were used as initial conditions for the reduction process.

Thus, two sets of coefficients were obtained (Tables 1 and 2): all possible 12 transport rate constants for oxidation and nine constants for reduction (without illumination); as in the latter case, there are no transitions from the first to the second, third, and fourth states (i.e., *k*_12_, *k*_13_, *k*_14_ = 0).

**Table 1:** Rate constants for oxidation process in RC. Exposure time 90 s, excitation light intensity 0.2–6 mW/cm^2^

*I*, mW/sm^2^	*k*_12_, s^−1^	*k*_21_, s^−1^	*k*_23_, s^−1^	*k*_32_, s^−1^	*k*_34_, s^−1^	*k*_43_, s^−1^	*k*_13_, s^−1^	*k*_31_, s^−1^	*k*_24_, s^−1^	*k*_42_, s^−1^	*k*_14_, s^−1^	*k*_41_, s^−1^
0.2	0.471	0.9995	5.01E−04	1.10E−06	1.10E−06	5.49E−02	0.0012	1.00E−06	6.16E−03	4.94E−02	1.00E−06	1.00E−06
0.5	1.5085	1.49375	7.70E−04	4.87E−02	2.17E−02	5.58E−04	0.02554	7.51E−04	1.59E−04	1.10E−06	1.00E−06	1.00E−06
1	3.94725	1.40738	6.01E−04	1.10E−06	1.10E−06	5.83E−02	0.01884	1.39E−04	5.35E−03	1.11E−01	0.007	6.89E−03
2	6.66	1.81312	0.07201	0.15489	7.07E−03	1.09E−05	0.01526	1.01E−05	1.06E−05	1.07E−05	0.024	1.03E−05
4	23.59125	6.22	0.05276	0.2087	1.08E−05	3.38E−03	0.09032	7.07E−03	3.79E−03	1.07E−05	0.01974	1.03E−05
6	40.81	5.01	0.07701	0.14101	2.50E−02	1.09E−05	1.00E−05	1.60E−02	1.06E−05	1.07E−05	0.01981	1.03E−05

**Table 2:** Rate constants for reduction process in RC. Exposure time 90 s, excitation light intensity 0.2–6 mW/cm^2^

*I*, mW/sm^2^	*k*_12_, s^−1^	*k*_21_, s^−1^	*k*_23_, s^−1^	*k*_32_, s^−1^	*k*_34_, s^−1^	*k*_43_, s^−1^	*k*_13_, s^−1^	*k*_31_, s^−1^	*k*_24_, s^−1^	*k*_42_, s^−1^	*k*_14_, s^−1^	*k*_41_, s^−1^
0.2	0	0.921	9.33E−02	5.60E−03	2.81E−02	1.32E−03	0	1.62E−02	3.20E−02	7.15E−03	0	1.00E−06
0.5	0	0.99287	1.05E−02	2.40E−02	1.10E−06	6.98E−05	0	9.39E−04	1.10E−06	1.40E−03	0	2.42E−03
1	0	0.15523	1.00E−06	1.10E−06	4.14E−04	4.50E−03	0	1.03359	1.99E−01	1.10E−06	0	1.00E−06
2	0	0.02543	0.00749	0.00568	1.10E−06	1.10E−06	0	1.00E−06	1.10E−06	1.10E−06	0	1.08E−06
4	0	1.091	0.00418	0.1914	1.10E−06	3.74E−03	0	1.69E−01	1.10E−06	1.10E−06	0	1.00E−06
6	0	0.0314	0.0085	0.0051	1.10E−06	7.45E−02	0	1.00E−06	1.10E−06	1.10E−06	0	1.02

**Fig. 6:**
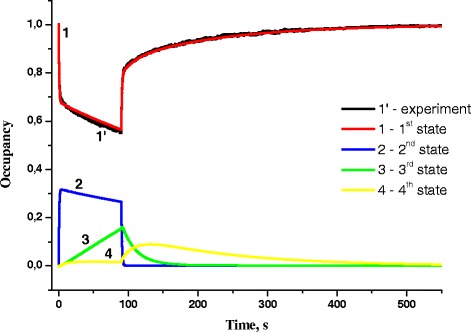
Occupancies of first, second, third, and fourth states of RC with light intensity 0.2 mW/cm^2^ and exposure time 90 s (*1’*—experiment, *1*—first state, *2*—second state, *3*—third state, *4*—fourth state)

**Fig. 7:**
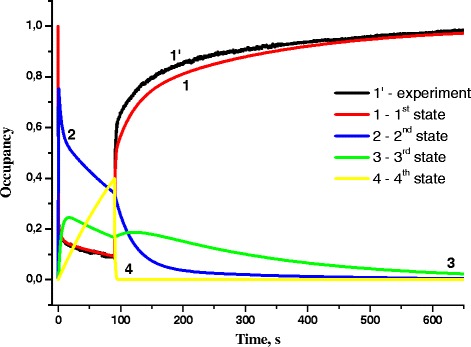
Occupancies of first, second, third, and fourth states of RC with light intensity 2 mW/cm^2^ and exposure time 90 s (*1’*—experiment, *1*—first state, *2*—second state, *3*—third state, *4*—fourth state)

**Fig. 8:**
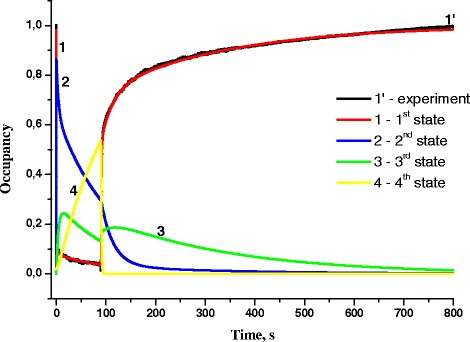
Occupancies of first, second, third, and fourth states of RC with light intensity 6 mW/cm^2^ and exposure time 90 s (*1’*—experiment, *1*—first state, *2*—second state, *3*—third state, *4*—fourth state)

The data allow to conclude that the processes of oxidation and reduction are characterized by different values of transport rate constants, since the structural changes in RC occur under the influence of external factors (illumination), whereas the relaxation (RC reduction) is an independent process; therefore, the values of transport rate constants for these two processes are different. Basing on these datasets, the occupancies of the first, second, third, and fourth states of RC were found for different photoexcitation modes (Figs. 5, 6, 7, and 8). As seen, both oxidation and reduction, within the error, are well consistent with the experimentally determined kinetics of the first state occupancy. So, we can trust the results obtained for the occupancies of the second, third, and fourth states at different modes of photoexcitation of RC system.

Let us consider the oxidation process at light intensity *I* = 0.2 mW/cm^2^: at first, the process of occupation of the second state from the first one occurs, and then, starting from *t* = 3 s, the occupancies of the third and fourth states increase with time, while the occupancies of the first and second states decrease. This means that during the excitement, the transitions to the third and fourth states occur not only from the first state but also from the second one (Fig. 5). After excitation light turning off, an abrupt drop of the second state occupancy from its maximum value and gradual reduction of the third state occupancy are observed. The fourth state occupancy increases for 40 s and then gradually decreases.

Under illumination by light with higher intensity *I* = 2 mW/cm^2^ (Fig. 6), the following processes are observed during oxidation: at first, the transitions from the first state to the second one occur; then, starting from 0.8 s, the third and fourth state occupancies increase due to transitions from the first and second states. Starting from *t* = 17 s, the occupancy of only fourth state increases, while the occupancies of other three states decreases. After excitation light turning off, an abrupt drop of the fourth state occupancy from its maximum value and gradual reduction of the second state occupancy are observed. The third state occupancy continues to increase for 33 s and then gradually decreases.

When light intensity is further heightened up to *I* = 6 mW/cm^2^ (Fig. 7), the oxidation starts with transitions from the first state to the second one; then, starting from *t* = 0.5 s, the third and fourth state occupancies increase due to transitions from the first and second states. Starting from *t* = 13 s, the occupancy of only fourth state increases, while the occupancies of other three states decrease. After excitation light turning off, an abrupt drop of the fourth state occupancy from its maximum value and gradual reduction of the second state occupancy are observed. The third state occupancy continues to increase for 35 s and then gradually decreases.

**Fig. 9:**
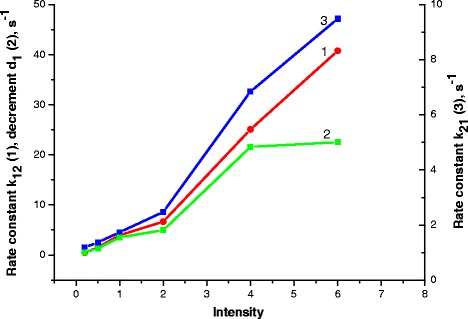
Oxidation process: *1*—rate constant *k*_12_, *2*—rate constant *k*_21_ (2), *3*—decrement *d*_1_, light intensity 0.2–6 mW/cm^2^, exposure time 90 s

An analysis of the dependences of oxidation at different light intensities revealed that the decrement *d*_1_ (applied in the procedure of approximation of experimental curves) qualitatively coincides with the rate constant *k*_12_ (Fig. 9). This suggests that these parameters characterize the fast electron processes in the absence of any conformations.

As seen from Fig. 9, the rate constant *k*_21_ which describes the electron return from the second to the first state during oxidation remains almost unchangeable at illumination with light intensities in the range of 0.2…2 mW/cm^2^ and grows with light intensity increasing from 2 to 6 mW/cm^2^. That is, the higher intensity, the shorter time the electron dwells on the quinone. This can be explained by the fact that in the quasi-classical approximation the electron staying on the quinone corresponds to a non-equilibrium state of the system: in this case, electromagnetic wave in the result of non-resonant interaction can excite oscillations in the electron subsystem of the particles of protein molecule of RC, which is partially ordered. And these oscillations, in turn, can excite oscillations in the nuclear subsystem of these particles. This will trigger local collective excitations—local phonons (fractions). Such collective excitations can facilitate the return of electron to the donor. This is the first process characterized by non-resonant interaction of electromagnetic wave with RC in non-equilibrium state. At the same time, an analysis of experimental curves shows that the increase of light intensity and exposure time results in a slower recovery of RC absorption ability after light turning off. This is the second process, and it is characterized by the influence of polarization on the internal processes of molecular electron transport. Thus, it can be assumed that during the oxidation of RC, two competing processes occur: one accelerates the recovery of absorption ability and another slows it down. Both these processes stimulate structural changes in RC.

Besides, an analysis of the recovery kinetics allows to draw the following conclusions: if the state occupancy at the moment of light turning off is the highest, then the rates of inverse transitions from this state also have the maximum values. In addition to returning from the second, third, and fourth states into the first one, the transitions between second, third, and fourth states are still possible. Similarly to the oxidation, in the reduction process, two competing processes also can be distinguished. The first process characterizes only the electron transport without changes in the structure of RC, corresponding to the relaxation of occupancy of the state which was most occupied at the moment of light turning off, while the second process reflects the kinetics of structural changes. During the oxidation and reduction, the processes of relaxation of electron transport with significantly different decrements are observed. This fact suggests that the RC system in a short time can get into states that significantly differ from each other in recovery time, which characterizes the structure of RC. The processes of transition from one state to another can proceed with anomalous diffusion (*E*{*x*_n_^2^} = *σ*^2^ < ∞, *E*{*t*_n_} = *t* = ∞). Such diffusion of structure can take place when the transition occurs by the mechanism of dispersion jumps provided by tunneling between traps separated by a low-energy barrier.

## Conclusions

The experimental data shows that the kinetics of absorption of RC solution during and after excitation are well approximated by a set of three exponential functions. The model of the slow electron transport and structural changes is constructed as a system of four electron-conformational states characterized by 12 and nine transport rate constants for oxidation and reduction, respectively. At the beginning, all RCs are the same, being in the first state with the electrons at the donor (thermo-adapted state). Under photoexcitation, some electrons are at the donor and other at the acceptor in the second, third, and fourth states. Each RC can transfer from one electron-conformational state into other during both oxidation and reduction.

Quantitative values of transport rate constants for oxidation and reduction of RC are found and the kinetics of the occupancies of the first, second, third, and fourth electron-conformational states of RC are calculated. A qualitative agreement between the experimental data for the occupancy of the first state, and the results of calculations for various modes of photoexcitement is shown.

The dependences of transport rate constants *k*_12_ and *k*_21_ for the oxidation process are determined: *k*_12_ is proportional to light intensity and *k*_21_ is almost invariable at light intensities of 0.2…2 mW/cm^2^, growing with intensity in the range of 2…6 mW/cm^2^. This is explained by the fact that during photoexcitation the RC is in non-equilibrium state under the impact of electromagnetic wave. It leads to structural changes in the RC.

The occurrence of two competing processes in the RC under photoexcitation is suggested. The first process accelerates the return of electron to the donor, and it is characterized by a non-resonant interaction of electromagnetic wave with the RC being in a non-equilibrium state. On the contrary, the second process slows down the recovery of absorption ability. It is characterized by the influence of polarization on the internal processes of molecular electron transport. Both the first and second processes cause structural changes in the RC. After light turning off, only the second process is observed.

It is established that if the occupancy of electron-conformational state of RC is maximum at the moment of light turning off, then the rates of inverse transitions from this state during the reduction also have maximal values.

## Abbreviations

B_A_: bacteriochlorophyll monomer
H: bacteriopheophytins
LDAO: lauryldimethylamine oxide
P: bacteriochlorophyll dimmer
Q_A_: primary quinone
Q_B_: secondary quinone
RC: reaction center
